# TREK-1 mediates isoflurane-induced cytotoxicity in astrocytes

**DOI:** 10.1186/s12871-017-0420-5

**Published:** 2017-09-05

**Authors:** Haiyun Guo, Zhengwu Peng, Liu Yang, Xue Liu, Yaning Xie, Yanhui Cai, Lize Xiong, Yi Zeng

**Affiliations:** 1 Department of Anesthesiology and Perioperative Medicine, Xijing Hospital, Fourth Military Medical University, Xi’an, 710032 China; 2 Department of Psychiatry, Xijing Hospital, Fourth Military Medical University, Xi’an, 710032 China; 3 Department of Neurobiology and Collaborative Innovation Center for Brain Science, Fourth Military Medical University, Xi’an, 710032 China

**Keywords:** TREK-1, Isoflurane, Cytotoxicity, Astrocyte, BDNF

## Abstract

**Background:**

There are growing concerns that anaesthetic exposure can cause extensive apoptotic degeneration of neurons and the impairment of normal synaptic development and remodelling. However, little attention has been paid to exploring the possible cytotoxicity of inhalation anaesthetics, such as isoflurane, in astrocytes. In this research, we determined that prolonged exposure to an inhalation anaesthetic caused cytotoxicity in astrocytes, and we identified the underlying molecular mechanism responsible for this process.

**Methods:**

Astrocytes were exposed to isoflurane, and astrocytic survival was then measured via LDH release assays, MTT assays, and TUNEL staining. TWIK-related potassium (K^+^) channel-1 (TREK-1) over-expression and knockdown models were also created using lentiviruses. The levels of TREK-1 and brain-derived neurotrophic factor (BDNF) were measured via Western blot and qRT-PCR.

**Results:**

Prolonged exposure to isoflurane decreased primary astrocytic viability in a dose- and time-dependent manner. Moreover, with prolonged exposure to isoflurane, the TREK-1 level increased, and the BDNF level was reduced. TREK-1 knockdown promoted the survival of astrocytes and increased BDNF expression following isoflurane exposure.

**Conclusions:**

Overdoses of and prolonged exposure to isoflurane induce cytotoxicity in primary astrocytes. TREK-1 plays an important role in isoflurane-induced cultured astrocytic cytotoxicity by down-regulating the expression of BDNF.

## Background

The rapid development and great achievements of modern general anaesthesia have guaranteed the safety of millions of surgeries every year. However, it has become widely accepted that these anaesthetics can induce widespread neurodegeneration and subsequent impairments of synaptogenesis [1–3]. Exposure to anaesthetics can specifically alter the morphology and density of dendritic spines in developing synapses and cause detrimental disruptions of neuronal actin cytoskeletons that are strongly age dependent [4, 5]. Additionally, significant and profound cognitive impairments have been observed in nonhuman primates following exposure to anaesthetics during a critical period of brain development [6].

To date, researches have been mainly focused on the effects of anaesthesia-induced neurotoxicity on neurons, but not astrocytes. However, given that the integrity and proper functioning of astrocytes are crucial to neuronal function, the neurological effects of anaesthetics on astrocytes should not be neglected [7]. As one of the most abundant types of cerebral cells, astrocytes are known to actively participate in brain functioning, development, and reactions to adverse conditions [8, 9]. Previous studies have demonstrated that isoflurane can negatively affect the maturation and growth of cultured astrocytes and cause tremendous cellular disturbances [10, 11]. Specifically, new evidence has revealed that application of 1.4% isoflurane for 4 h induces degenerative cytoskeletal changes in astrocytes but does not affect their survival, motility, or proliferation [7]. However, recent preliminary researches indicate that isoflurane can cause significant apoptotic changes in developing oligodendroglia in vivo [12]. Additionally, because the dosages and durations of exposure to anaesthetics vary between different surgeries, patients may occasionally suffer from an overdose or prolonged isoflurane exposure. Therefore, whether isoflurane can cause extensive impairments of neural cells, and especially astrocytes, with excessive exposure time and concentrations needs to be thoroughly examined.

TWIK-related potassium (K^+^) channel-1 (TREK-1) is a potassium channel that is located in the cytomembrane and involved in regulating the neuronal action potential duration, the resting membrane potential, and transmitter release [13]. Previous studies have reported that TREK-1 is an important target of the action for volatile anaesthetics and plays a dual role in various brain diseases [14–16]. Certain results have demonstrated that the opening of TREK-1 can protect neurons against glutamate excitotoxicity during brain ischaemia [17, 18], and other studies have demonstrated that the neuroprotective effects of sevoflurane preconditioning are significantly attenuated with TREK-1 knockdown [19, 20]. In addition, the authors of other studies have proposed that the TREK-1 gene is associated with responses to antidepressants in humans and a depression-resistant phenotype in rodents [21, 22]. However, the role of TREK-1 in isoflurane-induced astrocytic impairment has not been determined, which inspires us to conduct research to address this question.

In the present study, we observed that overdoses of and prolonged exposures to isoflurane could induce astrocytic injury in vitro, which hadn’t been investigated before. Next, we found that TREK-1 participated in the process of isoflurane-induced astrocytic injury. Specifically, the over-expression of TREK-1 exacerbated the cytotoxicity, and the inhibition of TREK-1 alleviated this isoflurane-induced injury to astrocytes. We thus believe that TREK-1 is a potential target for interventions aiming to relieve isoflurane-induced cultured astrocytic cytotoxicity.

## Methods

### Primary astrocyte cultures

Primary astrocyte cultures were prepared from postnatal day 1–2 C57BL/6 J Mice provided by the Experimental Animal Center of Fourth Military Medical University. Briefly, the cerebral cortices of pups (*n* = 8 per culture) were rapidly excised and transferred into Dulbecco’s modified Eagle’s medium (DMEM, HyClone, Logan, UT, USA) on ice. Next, the tissues were digested with 0.1% trypsin for 10 min at 37 °C in a sterile dish after the meninges were removed under a microscope. Trypsin digestion was terminated via the addition of DMEM supplemented with 10% fetal bovine serum (FBS, HyClone). The supernatants were subsequently centrifuged at 800 rpm for 20 min and re-suspended in DMEM with 10% FBS (complete culture medium). The cells were then plated in Poly-D-lysine-coated (0.01%) flasks (Invitrogen, Carlsbad, CA, USA) containing complete culture medium and maintained for 7 days to allow for growth and proliferation to 95% confluence. The medium was replaced every four days. Confluent astrocytes were shaken for 19 h at 180 rpm at 37 °C and then washed with sterile phosphate-buffered saline (PBS) to remove oligodendrocytes. Afterwards, the astrocytes were trypsinized and re-plated. These cells were used within 3 passages in all in vitro experiments.

### Isoflurane exposure

The medium in the cell plates with astrocytes was replaced with fresh complete medium, and the plates were transferred to identical humidified, airtight chambers (Billups-Rothenberg, Del Mar, CA, USA). The control group was treated with 21% O_2_, 5% CO_2_ and 74% N_2_, and the experimental groups were treated with control gas mixed with homologous concentrations of isoflurane. During the isoflurane exposure, the chambers were placed in an incubator to maintain the temperature at 37 °C. The concentration of isoflurane was measured every 3 h with an agent analyser (Ohmeda 5250 RGM, Louisville, CO, USA). The treatment was terminated when the cell plates were removed from the chambers and the culture medium was replaced with fresh complete medium. Isoflurane was again added to the chambers every 3 h considering the volatilization characteristics of isoflurane to maintain a stable concentration in the cultures. And we evaluated the potential hypoxia effects by detecting the concentration of O_2_ using agent analyser (Ohmeda 5250 RGM), which maintained at approximate 21% after 3 h after isoflurane exposure.

### 3-(4,5-dimethylthiazol-2-yl)-2,5-diphenyltetrazolium bromide (MTT) assay

The MTT assay was used to determine astrocytic viability. For this assay, astrocytes were plated on 96-well plates at 1000 cells per well. One day after plating, the astrocytes were exposed to isoflurane. And MTT (Sigma-Aldrich, St. Louis, MO, USA) was added to each well to yield a concentration of 0.5 μg/μl. The medium was removed from the wells after incubation for 3 h at 37 °C. Next, 150 μl of dimethyl sulfoxide (Sigma-Aldrich) was added to each well, and the plates were read using an absorbance plate reader (Tecan, Grodig, Austria) at a wavelength of 570 nm.

### Lactate dehydrogenase (LDH) release analysis

LDH activity was determined using the commercial LDH-Cytotoxicity Colorimetric Assay Kit (BioVision, Milpitas, CA, USA) according to the manufacturer’s protocol. After treatment with different doses of isoflurane, 50 μl of the supernatant was transferred to a fresh 96-well plate and mixed with 50 μl of the reaction mixture. After incubation for 30 min at room temperature, the absorbance was measured at 490 nm with an absorbance plate reader (Tecan).

### Terminal deoxynucleotidyl transferase dUTP nick end labelling (TUNEL) staining

After treatment with different doses of isoflurane, the primary cultured astrocytes were washed with PBS, fixed in 4% paraformaldehyde (Sigma-Aldrich) for 1.5 h, and permeabilized in 0.15% sodium citrate (Sigma-Aldrich) and 0.15% Triton™X-100 (Sigma-Aldrich) for 5 min at 4 °C. TUNEL staining was then performed using the In Situ Cell Death Detection Kit (Roche, Mannheim, Germany). Briefly, slides were incubated in 50 μl of TUNEL reaction mixture for 60 min at 37 °C in a humidified atmosphere in the dark. The cells were then stained with 4′,6-diamidino-2-phenylindole (DAPI, 1 μg/ml, Sigma-Aldrich) for 5 min to determine the total cell numbers, after which they were visualized under a fluorescence microscope (Olympus, Tokyo, Japan). The numbers of positive cells were measured in five fields per slide.

### Western blot analysis

After exposure to isoflurane, cells were harvested and lysed in extraction buffer (Thermo Scientific, Lafayette, CO, USA) containing a complete protease inhibitor (1%, Thermo Scientific). The concentration of proteins was detected via a BCA protein assay kit (Thermo Scientific). The same amounts of protein were separated on sodium dodecyl sulfate polyacrylamide gels and transferred to 0.22-μm polyvinylidene fluoride membranes (Millipore, Bedford, MA, USA) for 2 h, followed by blocking with skim milk (5%). The membranes were then incubated with the appropriate primary antibody, such as rabbit polyclonal antibody to TREK-1 (1:200, Abcam, Cambridge, MA, USA), rabbit monoclonal antibody to BDNF (1:2500, Abcam) or rabbit monoclonal antibody to β-tubulin (1:1000, Abcam) at 4 °C for 12 h. After three washes, the membranes were incubated with goat anti-rabbit secondary antibodies (1:10,000, Abcam) for 2 h. After the membranes were washed again, bands were detected using a chemiluminescent horseradish peroxidase substrate (Millipore). The band intensities were analysed using Quantity One software 5.0 (Bio-Rad, La Jolla, CA, USA).

### Quantitative reverse transcription polymerase chain reaction (qRT-PCR)

Astrocytic RNA was extracted with an RNA extraction kit (TaKaRa, Otsu, Japan) after exposure to isoflurane. The same amounts of total RNA from the astrocytes were reverse transcribed using PrimeScript RT Master Mix (TaKaRa) under standard conditions. Following reverse transcription, qRT-PCR reactions were performed with SYBR Premix Ex Taq (TaKaRa). The primer sequences were as follows (forward/reverse): TREK-1 (TCTGGTGGGCTTGTGGTTC/AGGGGAGGGGATAGGTGAGA), BDNF (GATTCGGGCCACTTGGAGTTA/TGGAGCAACACCAGGCAGAC), and GAPDH (AAATGGTGAAGGTCGGTGTGAAC/CAACAATCTCCACTTTGCCACTG). GAPDH was adopted as an internal control.

### In vitro TREK-1 silencing and over-expression models

Lentiviruses containing either TREK-1 siRNA or the TREK-1 gene were purchased from Invitrogen. Astrocytes (5 × 10^5^) were plated onto 100-mm plates, and one day later, the lentiviruses were added to the astrocytes at a multiplicity of infection of 50 with 8 mg/ml polybrene (Sigma-Aldrich) for 3 days. Next, the lentiviruses were discarded, and the astrocytes were trypsinized and plated in selection medium with 5 μg/μl blasticidin (Invitrogen) for 4 days. A scrambled-siRNA and a blank vector were used as negative controls, and the TREK-1 silencing and over-expression models were validated using qRT-PCR and Western blotting.

### Statistical analysis

The results are reported as the mean ± SEM. Student’s t-test, one-way analysis of variance (ANOVA), and two-way ANOVA followed by Bonferroni’s multiple comparisons test were used for the statistical analyses and were performed with Prism 5 (GraphPad Software). *P* values <0.05 were considered statistically significant.

## Results

### Isoflurane overdoses and prolonged exposure elicited toxic effects on astrocytes

**Fig. 1:**
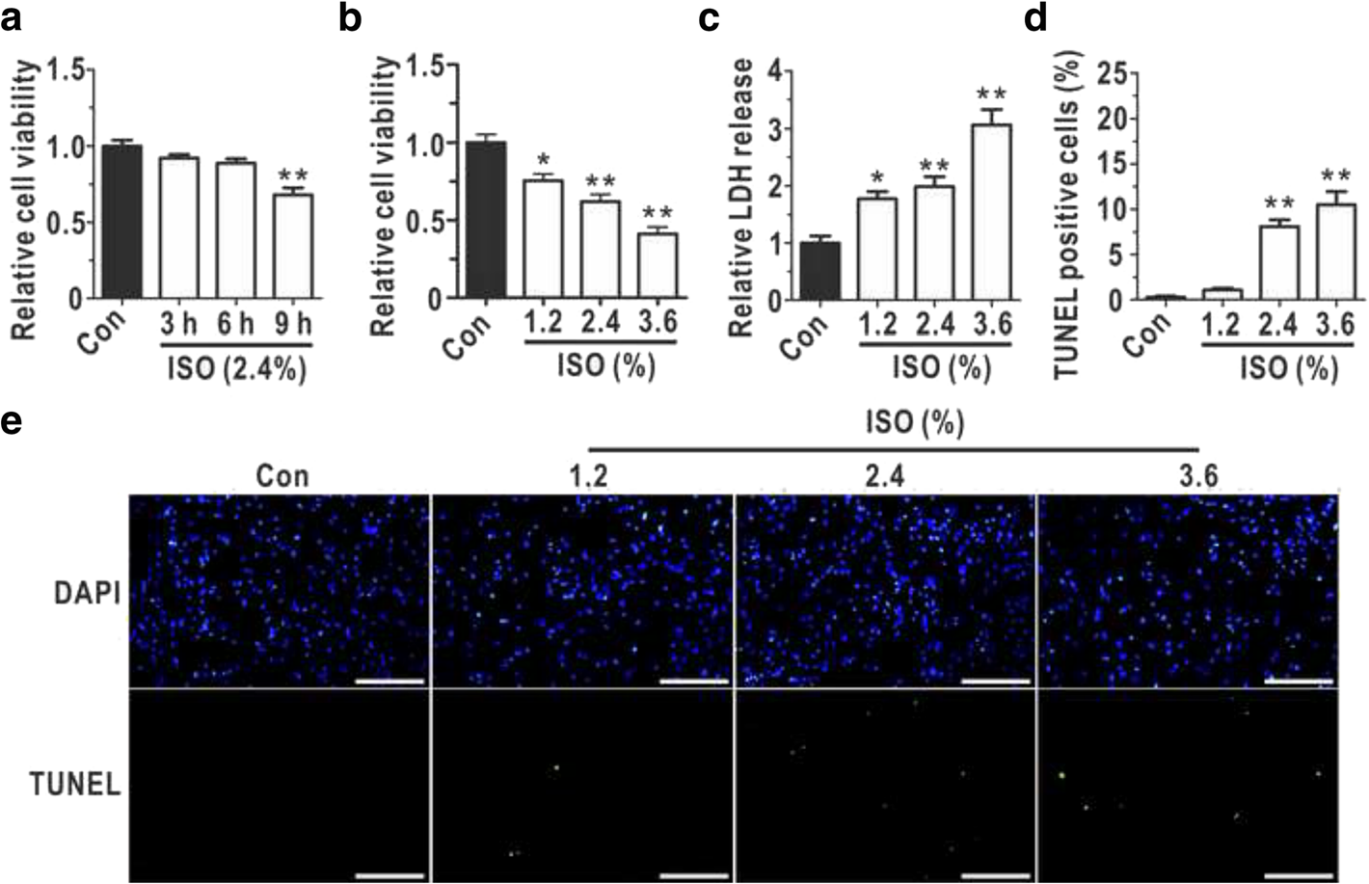
Prolonged isoflurane exposure induced dose-dependent toxic effects in cultured astrocytes. **a** Cell viability was measured with MTT assays following exposure to isoflurane for different hours at 2.4%, and the relative cell viabilities of the experimental groups were normalized to that of the control group (*N* = 4). **b** The cell viability was determined with MTT assays following exposure to different doses isoflurane for 9 h (*N* = 5). **c** LDH release was quantitatively measured as a biomarker of cellular cytotoxicity and cytolysis, and the relative LDH release levels in the experimental groups were normalized to that of the control group after isoflurane exposure for 9 h (*N* = 5). **d** The percentages of TUNEL-positive astrocytes after isoflurane exposure for 9 h are shown (*N* = 3). **e** The graphs display representative data from three independent experiments (*N* = 3). Bar = 200 μm. ISO, isoflurane. The data are presented as the mean ± SEM. ^*^ *P* < 0.05, ^**^ *P* < 0.01. The *P* values were determined by one-way ANOVA (**a**, **b**, **c** and **d**) with correction for multiple comparisons

To test whether extended exposure to isoflurane interfered with astrocytic viability, primary astrocytes were treated with 2.4% isoflurane for different hours (3 h, 6 h and 9 h) using MTT analysis (Fig. 1a). As shown in Fig. 1a, 9 h isoflurane exposure induced a significant cytotoxicity on astrocytes, and the following experiments were carried out using 9 h isoflurane exposure. The MTT assays (Fig. 1b) revealed that the astrocytic viability was reduced with increasing doses of isoflurane, and the astrocytic LDH release was found to be increased in a dose-dependent manner (Fig. 1c). Additionally, we observed that apoptosis played a role in the isoflurane-induced astrocytic cytotoxicity (Fig. 1e) and that the apoptosis ratio increased after exposure to isoflurane (Fig. 1d). These data verified that isoflurane overdoses and prolonged exposure to isoflurane treatment induced astrocytic cytotoxicity.

### TREK-1 and BDNF in cultured astrocytes exhibited corresponding changes following isoflurane exposure

**Fig. 2:**
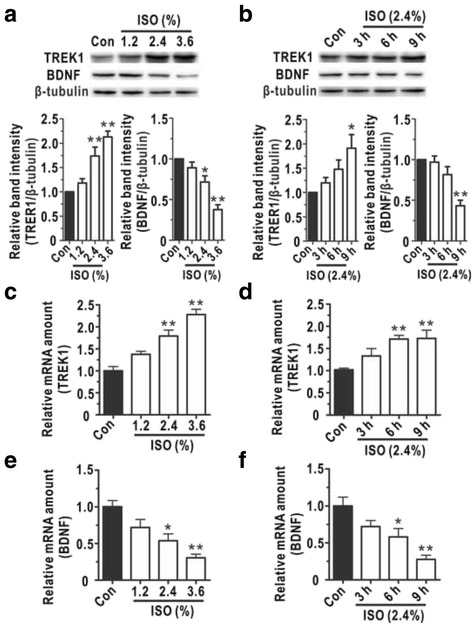
The expression of related proteins exhibited time- and dose-dependent changes. Western blotting was performed to analyse the expression of TREK-1 (**a**, *N* = 4) and BDNF (**b**, *N* = 4) in astrocytes following different isoflurane doses and exposure time. Quantitative reverse transcription-polymerase chain reaction (qRT-PCR) assays were used to calculate the relative TREK-1 (**c** and **d**, *N* = 5) and BDNF (**e** and **f**, *N* = 4) mRNA levels in astrocytes following different isoflurane doses and exposure time. Data are presented as relative to Con group. (**a**, **c** and **e**) Astrocytes were treated with different doses of isoflurane (0%, 1.2%, 2.4%, and 3.6%) for 9 h. (**b**, **d** and **f**) Astrocytes were treated with 2.4% isoflurane for different durations (0, 3, 6, and 9 h). ISO, isoflurane. The data are presented as the mean ± SEM. ^*^ *P* < 0.05, ^**^ *P* < 0.01. The *P* values were determined by one-way ANOVA (**a**, **b**, **c**, **d**, **e** and **f**) with correction for multiple comparisons

We next explored the mechanism by which isoflurane overdoses and prolonged isoflurane exposure elicited astrocytic cytotoxicity. Because TREK-1 is critical for the action of general volatile anaesthetics, we detected its expression level via Western blotting and qRT-PCR. Figure 2a demonstrated that the expression of TREK-1 was elevated in a dose- and time-dependent manner after isoflurane treatment. Because BDNF signalling is thought to be an important neuroprotective agent in brain injury recovery [23, 24], the BDNF levels were also detected to determine whether this neuroprotective molecule was translationally modulated by isoflurane exposure. With increasing isoflurane doses (0%, 1.2%, 2.4%, and 3.6%) and prolonged exposure time (0, 3, 6, and 9 h), the expression levels of BDNF decreased compared with those in the control group (Fig. 2b). In addition, we used qRT-PCR to detect the mRNA levels of TREK-1 and BDNF after isoflurane exposure. Similar to what was observed for the protein levels, the mRNA level of TREK-1 was decreased (Fig. 2c and d), and the mRNA level of BDNF was increased (Fig. 2e and f). These results suggested that isoflurane up-regulated the expression of the detrimental TREK-1 and down-regulated the expression of the beneficial BDNF in a dose- and time-dependent manner.

### The over-expression of TREK-1 contributed to isoflurane-induced astrocytic cytotoxicity

**Fig. 3:**
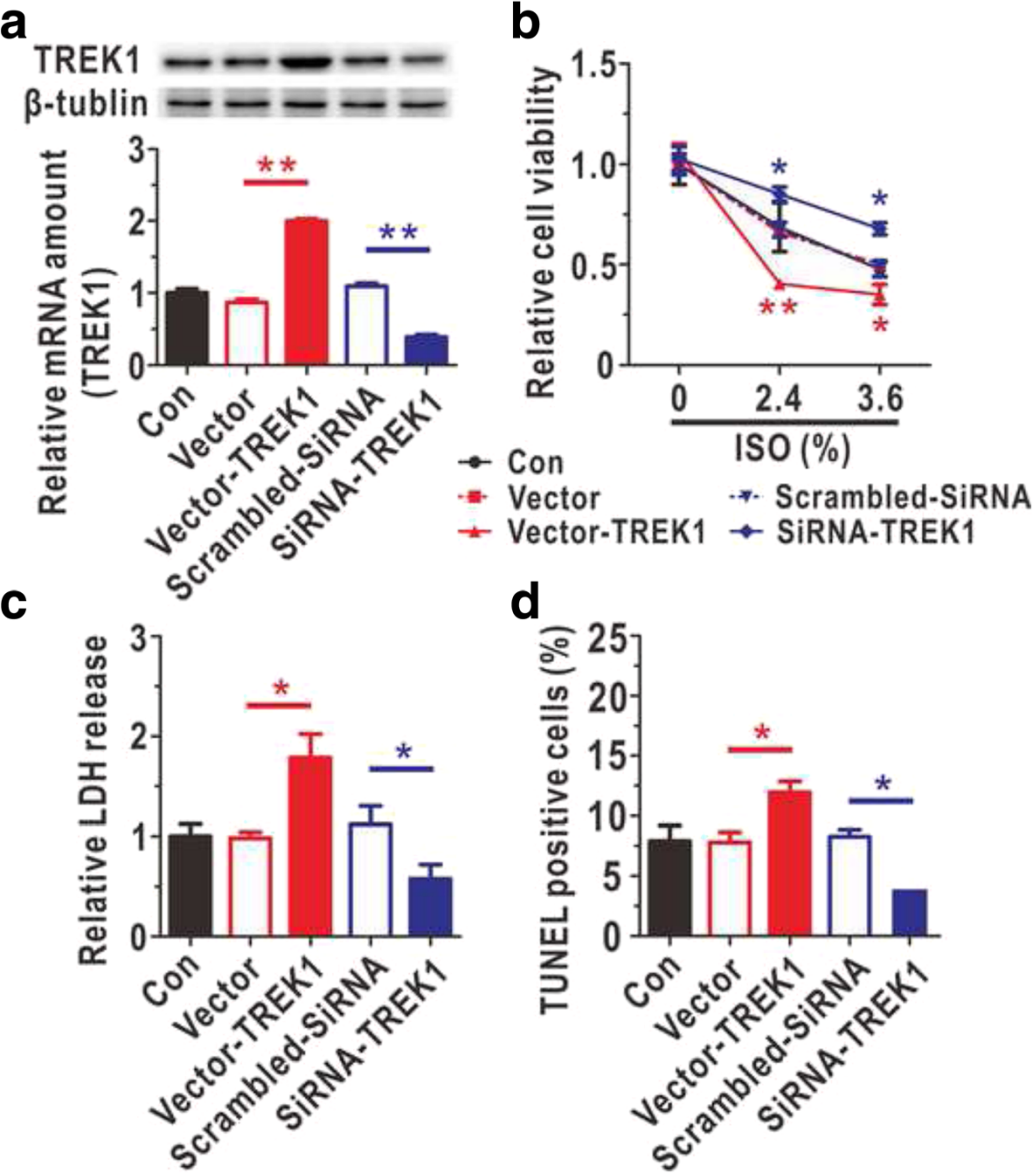
The over-expression of TREK-1 aggravated isoflurane-induced astrocytic cytotoxicity. **a** Verification of the astrocytic TREK-1 over-expression and knockdown models by Western blotting (*N* = 4) and qRT-PCR (*N* = 5). **b** Astrocytic viabilities were detected in the TREK-1 over-expression and knockdown models using MTT assays after treatment with different isoflurane doses (0%, 2.4%, and 3.6%) with an exposure duration of 9 h (*N* = 5). Data are presented as relative to Con group. **c** The cytotoxicity induced by isoflurane (2.4%) exposure for 9 h was verified via LDH assay (*N* = 5). Data are presented as relative to Con group. **d** The percentages of TUNEL-positive cells after 9 h of isoflurane exposure (2.4%) were determined in the astrocytic TREK-1 over-expression and knockdown models (*N* = 3). ISO, isoflurane. The data are presented as the mean ± SEM. ^*^ *P* < 0.05, ^**^ *P* < 0.01. The *P* values were determined by one-way ANOVA (**a**, **c** and **d**) or two-way ANOVA (**b**) with correction for multiple comparisons

To investigate whether TREK-1 contributed to isoflurane-induced injury, we constructed astrocytic TREK-1 over-expression and knockdown models using lentiviruses and verified the models by qRT-PCR and Western blotting (Fig. 3a). One week after lentiviral infection, the cytotoxic effects of 0%, 2.4%, and 3.6% isoflurane treatments for 9 h were measured. When the concentration of isoflurane was 0%, the over-expression and knockdown alone did not affect the viability of astrocytes (Fig. 3b). Besides, the over-expression of TREK-1 significantly decreased astrocytic viability after exposure to 2.4% or 3.6% isoflurane, and the knockdown of TREK-1 elicited the opposite effect (Fig. 3b). To further investigate this phenomenon, LDH assays and TUNEL staining were performed. We observed that the knockdown of TREK-1 protected against isoflurane-induced cytotoxicity following exposure to 2.4% isoflurane for 9 h (Fig. 3c and d). These results demonstrated that the inhibition of TREK-1 expression protected the astrocytes from isoflurane-induced damage and that the over-expression of TREK-1 exacerbated the isoflurane-induced astrocytic injury.

### TREK-1 negatively regulated BDNF expression during isoflurane-induced astrocytic toxicity

**Fig. 4:**
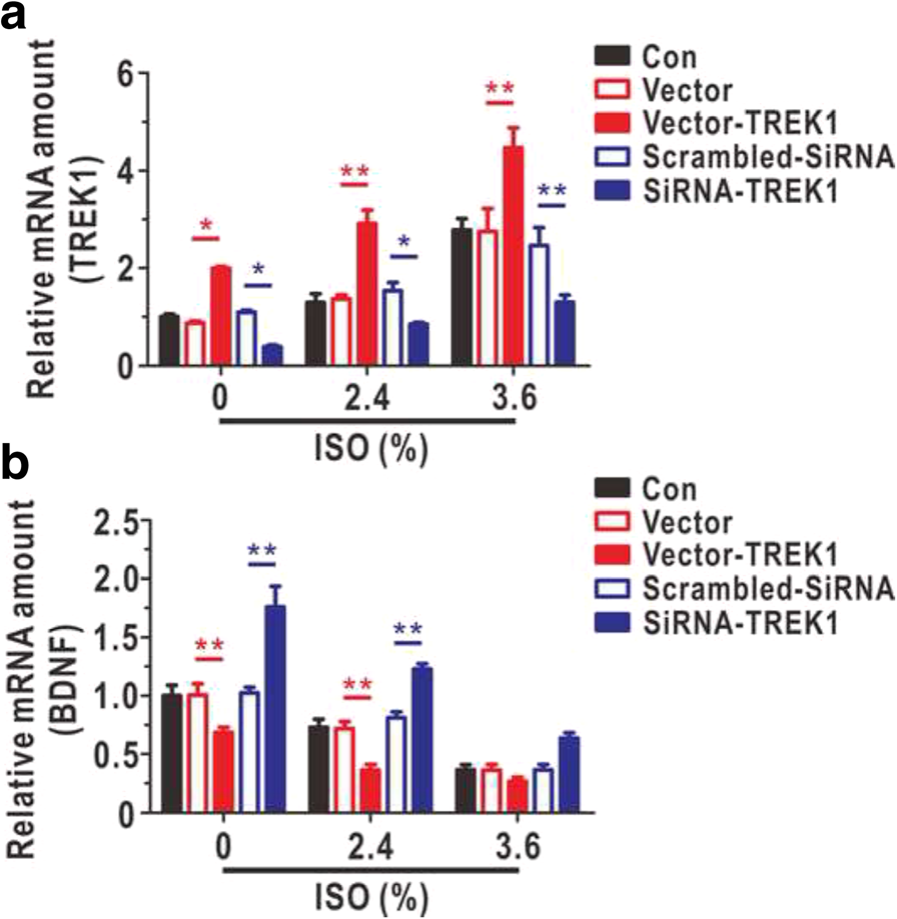
Regulation of astrocytic TREK-1 influenced the expression of BDNF in cultured astrocytes after isoflurane exposure. The TREK-1 (**a**, *N* = 5) and BDNF (**b**, *N* = 5) mRNA levels were analysed by qRT-PCR in TREK-1 overexpression and knock-down models. **a** and **b** were treated with different doses of isoflurane (0%, 2.4%, and 3.6%) for 9 h. ISO, isoflurane. Data are presented as relative to the values of 0% isoflurane in Con group. The data are presented as the mean ± SEM. ^*^ *P* < 0.05, ^**^ *P* < 0.01. The *P* values were determined by two-way ANOVA (**a** and **b**) with correction for multiple comparisons

The TREK-1 expression levels were significantly elevated with increasing doses of isoflurane, as assessed by qRT-PCR (Fig. 4a). Moreover, as illustrated in Fig. 4b, we found that the knockdown of TREK-1 increased BDNF expression and that the over-expression of TREK-1 decreased the level of BDNF with increasing doses of isoflurane. To our surprise, the knockdown of TREK-1 in astrocytes induced a significant increase in the expression of BDNF after treating with 2.4% isoflurane compared with expression in the vector group, but this increase was nonsignificant with the administration of 3.6%, possibly because the inhibitory effects of isoflurane at this high dose were so strong that the knockdown of TREK-1 could not rescue the expression of BDNF. These data demonstrated that TREK-1 regulated the BDNF pathway during isoflurane-induced astrocytic toxicity.

## Discussion

The functional integrity of astrocytes is critical for normal brain functions, including synaptic activity, energy metabolism, and extracellular ion homeostasis [9]. Astrocytes respond to a range of cerebral disorders, such as cerebral ischaemia, neurodegeneration, infection, migraine, and cerebral oedema [25–27]. Previous experiments demonstrate that isoflurane exposure disturbs mature astrocytes and interferes with their ability to support neuronal growth [10, 11]. Additionally, isoflurane treatment has previously been found to alter the cytoskeletons of astrocytes but not to significantly induce cell death [7]. Our results seem to contradict the results of previous experiments. However, the differences between the previous and present experiments in terms of isoflurane exposure times (4 h vs. 9 h, respectively) and concentrations (1.4% vs. 3.6%, respectively) could have resulted in mechanistic changes that induce significant differences in cell viability. It is true that 9 h of anaesthesia is rare in surgery, but trauma patients who need complex surgery do have an extended surgery time, and extended time is sometimes inevitable in certain other cases. In addition, no relevant studies have been performed to determine whether prolonged anaesthesia induces injury to astrocytes, which is the major purpose of this study. It should be mentioned that in vivo studies are needed to further confirm isoflurane exposure could induce astrocytic injury, and the survival of astrocytes may be improved due to the support from adjacent neurons in vivo after isoflurane stress.

TREK-1 is a signal integrator that responds to a series of physiological and pathological inputs and therefore contributes to the cellular processes associated with volatile general anaesthesia [15, 28]. Recently, the inhibition of TREK-1 in mice has revealed its important roles in a variety of neuronal processes, including neuroprotection, pain perception, and depression [17]. Moreover, certain studies have demonstrated that TREK-1 can be up-regulated during chronic and acute brain ischaemia and that TREK-1 activity is involved in neuronal and astrocytic survival [19, 29]. In our experiments, we found that TREK-1 played an important role in the astrocytic injury induced by isoflurane overdoses and prolonged isoflurane anaesthesia. Moreover, we verified that the inhibition of TREK-1 rescued astrocytes from isoflurane-induced injury.

To investigate the downstream mechanism responsible for the neuroprotective effects elicited by TREK-1 inhibition in isoflurane-induced astrocytic cytotoxicity, we analysed the association between TREK-1 and BDNF. BDNF is known as a key regulator of neural circuit function that is involved in cell differentiation, proliferation and modulation of synapse formation and function, and that contributes to learning, long-term memory consolidation, and repair of the brain and spinal cord following injury [30–33]. One study reports that activation of the BDNF pathway controls the morphology of cytoskeletal changes in astrocytes [34]. Moreover, previous research demonstrates that the synthesis of BDNF is enhanced following the inhibition of TREK-1 [35]. In the present study, we also found that the knockdown of TREK-1 increased the level of BDNF following isoflurane-induced astrocytic injury, and we thus inferred that BDNF participated in the protective effects associated with decreased TREK-1 levels following isoflurane-induced astrocytic cytotoxicity.

## Conclusion

The present research demonstrated that prolonged exposure to isoflurane induced cytotoxicity in primary astrocytes in a dose-dependent manner. Furthermore, increasing isoflurane doses and exposure time could induce TREK-1 up-regulation and consequently decrease BDNF expression. Additionally, we found that the knockdown of TREK-1 expression increased astrocytic viability following isoflurane-induced damage and that the over-expression of TREK-1 induced the opposite effect. The inhibition and over-expression of TREK-1 tended to up- and down-regulate, respectively, the expression of BDNF. Future work needs to explore the mechanism by which TREK-1 channels act as underlying moderators of the expression of BDNF, considering that TREK-1 channels could be a potential therapeutic target for protecting cultured astrocytes and eliminating isoflurane-induced toxicity.

## Abbreviations

BDNF: Brain-derived neurotrophic factor
LDH: Lactate dehydrogenase
MTT: 3-(4,5-dimethylthiazol-2-yl)-2,5-diphenyltetrazolium bromide
qRT-PCR: Quantitative reverse transcription polymerase chain reaction
TREK: TWIK-related potassium (K^+^) channel
TUNEL: Terminal deoxynucleotidyl transferase dUTP nick end labelling
